# Multiplex Real-Time PCR Diagnostic of Relapsing Fevers in Africa

**DOI:** 10.1371/journal.pntd.0002042

**Published:** 2013-01-31

**Authors:** Haitham Elbir, Mireille Henry, Georges Diatta, Oleg Mediannikov, Cheikh Sokhna, Adama Tall, Cristina Socolovschi, Sally J. Cutler, Kassahum D. Bilcha, Jemal Ali, Dayana Campelo, Steven C. Barker, Didier Raoult, Michel Drancourt

**Affiliations:** 1 Aix Marseille Université, URMITE, UMR63, CNRS 7278, IRD 198, Inserm 1095, Marseille, France; 2 URMITE, UMR IRD 198 CNRS 7278, Dakar, Senegal; 3 Institute Pasteur, Dakar, Senegal; 4 School of Health, Sports and Bioscience, University of East London, London, United Kingdom; 5 College of Medicine and Health Sciences, University of Gondar, Gondar, Ethiopia; 6 Parasitology Section, School of Chemistry and Molecular Bioscience, The University of Queensland, Brisbane, Australia; Oxford University Clinical Research Unit, Viet Nam

## Abstract

**Background:**

In Africa, relapsing fever borreliae are neglected arthropod-borne pathogens causing mild to deadly septicemia and miscarriage. The closely related *Borrelia crocidurae, Borrelia duttonii, Borrelia recurrentis* and *Borrelia hispanica* are rarely diagnosed at the species level, hampering refined epidemiological and clinical knowledge of the relapsing fevers. It would be hugely beneficial to have simultaneous detection and identification of *Borrelia* to species level directly from clinical samples.

**Methodology/Principal Findings:**

We designed a multiplex real-time PCR protocol targeting the 16S rRNA gene detecting all four *Borrelia*, the *glpQ* gene specifically detecting *B. crocidurae*, the *recN* gene specifically detecting *B. duttonii/B. recurrentis* and the *recC* gene specifically detecting *B. hispanica*. Compared to combined 16S rRNA gene and *flaB* gene sequencing as the gold standard, multiplex real-time PCR analyses of 171 *Borrelia*-positive and 101 *Borrelia*-negative control blood specimens yielded 100% sensitivity and specificity for *B. duttonii/B. recurrentis* and *B. hispanica* and 99% sensitivity and specificity for *B. crocidurae*.

**Conclusions/Significance:**

The multiplex real-time PCR developed in this study is a rapid technique for both molecular detection and speciation of relapsing fever borreliae from blood in Africa. It could be incorporated in point-of-care laboratory to confirm diagnosis and provide evidence of the burden of infection attributed to different species of known or potentially novel relapsing fever borreliae.

## Author Summary

Four cultured *Borrelia* species are responsible for relapsing fever in Africa. Three species (*Borrelia crocidurae, Borrelia duttonii, Borrelia hispanica*) are transmitted by ticks, whereas *Borrelia recurrentis* is transmitted by the body lice. These *Borrelia* species result in febrile infection mimicking malaria with varying severity, but particularly devastating during pregnancy where infection will often cause miscarriage. The lack of comprehensive laboratory tools for detection and speciation of these borreliae limits both medical management of patients and knowledge of the epidemiology of relapsing fevers in Africa. Based on genome analysis, we develop herein a multiplex real-time PCR assay targeting the 16S rRNA gene detecting all four borreliae, the *glpQ* gene detecting *B. croidurae*, the *recN* gene detecting *B. duttonii/B. recurrentis* and *recC* gene detecting *B. hispanica*. Compared to gold standard, this multiplex real-time PCR assay yielded 100% sensitivity and specificity for *B. duttonii/B. reccurentis* and *B. hispanica* and 99% sensitivity and specificity for *B. crocidurae*, when applied to 398 blood specimens. These findings provide the proof-of-concept that multiplex real-time PCR is a new tool for diagnosis of relapsing fever borreliae in Africa.

## Introduction

In Africa, relapsing fevers are neglected febrile infections caused by *Ornithodoros* spp. tick-borne borreliae (*Borrelia crocidurae*, *Borrelia hispanica* and *Borrelia duttonii*) and the *Pediculus humanus* louse-borne *Borrelia recurrentis* [1], [2]. Also, poorly characterized, yet uncultured new “*Borrelia mvumi*” species has been reported in acute patient's blood and *Ornithodoros porcinus* argasid ticks in Tanzania [3]. The relative geographic specificity of each *Borrelia* species has been challenged by coexistence of two species in the same region [4]. *B. hispanica* prevalence was reported to be 20.5% among febrile patients in Northwestern Morocco [5]; the prevalence of cases attributed to *B. crocidurae* among febrile patients is of 11 per 100 person-years in Senegal [1]. *B. duttonii* has been documented in Tanzania and *B. recurrentis* in Ethiopia [5], [6], [7], [8]. Relapsing fevers are of further concern in travelers returning from Africa in Europe [9], [10], [11].

Relapsing fevers are treatable infections but the severity of the disease ranges from asymptomatic to fatal if left untreated [12]. In Rwanda and in Tanzania the investigators found a 30% risk for pregnancy loss and a perinatal mortality rate of 15% [8], [13]. The prognosis depends in part on the causative species with the case-fatality ratio being higher for *B. recurrentis* infection than for the other infections [12]. However, the vast majority of patients are diagnosed on the basis of non-specific clinical features that overlap with those of malaria [4] and the poorly-sensitive, non-species specific microscopic observation of blood-borne *Borrelia* [1].

PCR-based tests have been therefore developed to improve the laboratory-based diagnosis of relapsing fevers in Africa [4]. In particular, real-time PCR targeting the 16S rRNA gene or the *glpQ* gene improved the sensitivity of the diagnosis when compared to microscopy [14], [15]. Also, we previously showed that PCR-sequencing intergenic spacers could be used for genotyping *B. crocidurae*, *B. duttonii* and *B. recurrentis* [16].

Here, we present the development and evaluation of a multiplex, quantitative real-time PCR detecting any relapsing fever *Borrelia* [14] and specifically *B. crocidurae*, *B. hispanica* and *B. duttonii/B. recurrentis* based on post-genomic analyses [16], [17]

## Materials and Methods

### Reference specimens and DNA extraction

*B. crocidurae* Achema strain, *B. recurrentis* A1 strain and *B. duttonii* Ly strain were grown in BSK-H medium (Sigma-Aldrich, Saint Quentin Fallavier, France) supplemented with heat-inactivated 10% rabbit serum (Eurobio, Courtaboeuf, France) before DNA extraction. *B. hispanica* DNA was directly extracted from two argasid ticks *Ornithodoros erraticus* sensu lato collected from Morocco. DNA was extracted from all specimens using QIAamp DNA Blood mini kit (QIAGEN, Hilden, Germany) according to the manufacturer's instructions. Reference identification of borreliae was made by combining the 16S rRNA gene and *flaB* gene sequencing [6], [18].

### Clinical specimens and DNA extraction

Total blood DNA was extracted from 21 blood specimens found positive by microscopy collected in 1994 from patients with relapsing fever in Addis Ababa, Ethiopia, 18 specimens collected in Mvumi, Tanzania [19], 9 specimens collected in 2011 in Bahir Dah, Highlands of Ethiopia [19] and 224 blood specimens collected from febrile patients from Ndiop and Dielmo villages in Senegal between 2008 and 2012 where *B. crocidurae* is endemic.

### Primers and probes design

Genome sequence of *B. duttonii* (GenBank accession number CP000976), *B. recurrentis* (GenBank accession number CP000993) and *B. crocidurae* (GenBank accession number CP003426.1) were downloaded from GenBank. Comparative genomic analyses were performed on the chromosomes in order to identify species-specific sequences. In addition, a 16SrRNA gene sequence-based system previously developed in our laboratory was used for *Borrelia* genus detection as previously described (14). Sequence alignments were performed using MULTALIN software for selection of each target sequence [20]. The primers and probes were constructed by using primer3 program at.http://frodo.wi.mit.edu/. Specificity of primers and probes were determined *in-silico*. Two different fluorescent dyes, VIC and FAM were used for labeling the probes.

### Real-time PCR

The single real-time PCR experiment was performed on Roche Lightcycler (RocheDiagnostic, Maylan, France). The amplification program included two initial holds at 50°C for 2 min and 95°C for 15 min, followed by 40 cycles consisting of 95°C for 30 seconds and 60°C for 1 minute. Five µL of extracted DNA, 0.5 µL of each primer (10 pmol) and 0.5 µL of probe (10 pmol) were added to the 10 µL Quantitative PCR Master mix (Quantitec, Qiagen) and the volume was adjusted to 15 µL by adding distilled water. The multiplex real-time PCR was performed using a Stratagene Mx3000P real-time thermocycler (Agilent, Courbevoie, France) by adding five microliters of extracted DNA, 0.5 µL of each primer (10 pmol) and 0.5 µL of each probe (10 pmol) [1 labeled with FAM and 1 labeled with VIC] to 12.5 µL of Quantitative PCR Master mix 2X (Quantitec) and the final volume was adjusted to 20 µL by adding distilled water. Negative control consisting of DNA-free water was included every 10 tested specimens.

### Real-time PCR specificity and sensitivity

**Figure 1:**
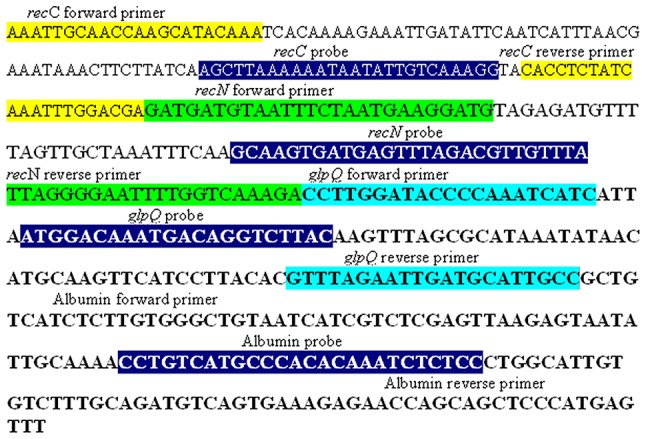
Sequence of the chimeric plasmid used as internal control in the real-time PCR. Yellow characters; *B. hispanica recC* gene sequence; green characters; *B. duttonii recN* gene sequence; pink character; *B. crocidurae glpQ* gene sequence; Red characters; albumine gene sequence; probes are in blue; Colored boxes contain the primer and probe sequences.

To assess the specificity of the real-time PCR systems developed herein, DNA extracted from Borrelia burgdorferi, Borrelia hermsii, Borrelia parkeri, Coxiella burnetii, Bartonella henselae, Rickettsia africae, Rickettsia felis and Tropheryma whipplei were incorporated into real-time PCR using the experimental conditions described above. In order to determine sensitivity, a puc 57 plasmid was constructed containing the human albumin gene, a 129-bp recN gene fragment from B. duttonii, a 122-bp recC gene fragment from B. hispanica and a 110-bp glpQ gene fragment from B. crocidurae (Invitrogen, Saint Aubin, France) (Figure 1). Tenfold serial dilutions of this constructed puc 57 plasmid were prepared equivalent to 10^7^ to 10^1^ Borrelia organisms.

### Ethics statement

This study was approved by the IFR48 Ethic Committee. All patients provided informed written consent.

## Results

### Species-specific primers and probes sequences

**Figure 2:**
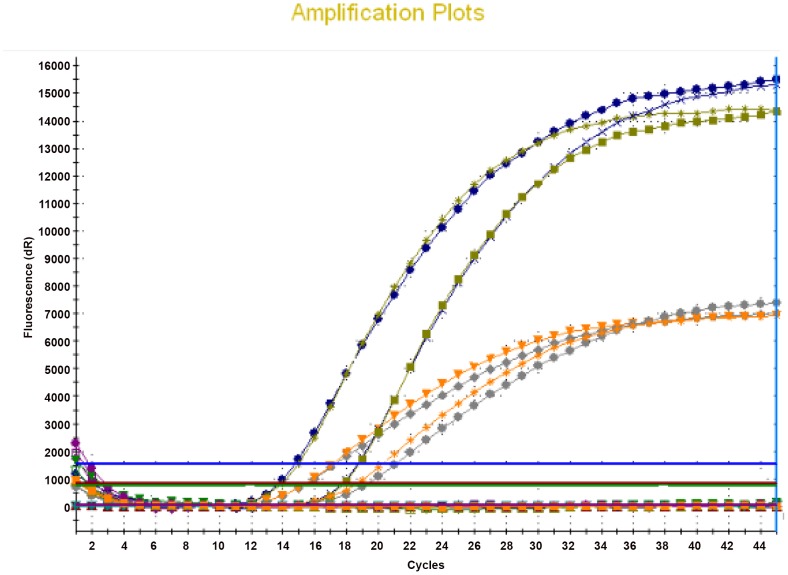
Fluorescence curves of four *Borrelia crocidurae* positive blood samples. Multiplex real-time PCR amplification and detection of the *Borrelia* genus-specific 16S rRNA gene (probe labeled with FAM fluorescent dye) and the *Borrelia crocidurae*-specific *glpQ* gene (probe labeled with VIC fluorescent dye).

As a specific target for *B. crocidurae*, we selected *glpQ* encoding glycerophosphodiester phosphodiesterase that is conserved among relapsing fever borreliae but absent from Lyme disease borreliae and additionally possesses a *B. crocidurae* specific 4-bp single nucleotide polymorphisms (SNPs). We selected the chromosomal *recN* gene encoding DNA repair ATPase for *B. duttonii/B. recurrentis*, that is conserved among the relapsing fever group and absent in the Lyme disease group borreliae, and furthermore exhibits a 5-bp specific SNP. For *B. hispanica*, we selected *recC* gene encoding exodeoxyribonuclease V, present in both relapsing fever and Lyme disease group borreliae and exhibiting 4-bp species-specific SNP. One probe specific for each of the three targeted regions was designed to span the region containing the SNPs. Sequences of the primers and probes are given in Table 1. Each set of species-specific primers and probe was first evaluated alone before being incorporated into a multiplex format. There was no difference in the amplification curves when comparing the single-target real-time PCR with multi-target real-time PCR assays. Figure 2 illustrates the results of these two experimental steps and shows that 16SrRNA gene probe labeled with FAM and *glpQ* gene probe labeled with VIC fluorescent dyes could both be detected in one PCR reaction performed simultanoiusly. Similar results were obtained with *recN* and *recC* labeled with VIC and 16SrRNA probe labeled with FAM.

**Table 1:** Primer and probe sequences used for multiplex real-time PCR.

Species	Primer/probes	Sequence 5′ to 3′
*B. crocidurae*	g*lpQ* forward primer	CCTTGGATACCCCAAATCATC
	g*lpQ* reverse primer	GGCAATGCATCAATTCTAAAC
	g*lpQ* MGB probe	6-FAM-ATGGACAAATGACAGGTCTTAC-NFQ
*B. duttonii/B. recurrentis*	*recN* forward primer	GATGATGTAATTTCTAATGAAGGATG
	*recN* reverse primer	TCTTTGACCAAAATTCCCCTAA
	*recN* probe	6-VIC- GCAAGTGATGAGTTTAGACGTTGTTTA-TAMRA
*B. hispanica*	*recC* forward primer	AAATTGCAACCAAGCATACAAA
	*recC* reverse primer	TCGTCCAAATTTGATAGAGGTG
	*recC* MGB probe	VIC-AGCTTAAAAAATAATATTGTCAAAGG-NFQ

### Real-time PCR specificity and sensitivity

In all experiments, negative controls remained negative. The cycle threshold (Ct) values for the constructed plasmid ranged from 18 (10^7^ copies) to 36 (100 copies) per 5 µL of plasmid dilution for *recN, recC* and *glpQ*. Based on these results, we used a Ct cutoff value of 36 for interpretation a clinical blood specimens as positive. The 16S rRNA probe detected all the *Borrelia*-positive specimens regardless of the species with Ct values ranging from 18 to 35. The *glpQ* assay designed to be specific for *B. crocidurae* did not amplify *B. duttonii* or *B. recurrentis* reference strains, 18 *B. duttonii*-positive blood samples, 30 *B. recurrentis*-positive blood samples, two *B. hispanica*-positive ticks, and other strains mentioned above. The *recN* assay for specific detection of *B. duttonii/B. recurrentis* did not detect *B. crocidurae, B. hispanica, B. burgdorferi* and other strains mentioned above. Likewise, the *recC* system specific for *B. hispanica* did not detect *B. crocidurae*, *B. duttonii/B. recurrentis*, *B. burgdorferi* and other strains mentioned above.

### Blood specimens

Human albumin used as a positive control was detected by real-time PCR in all tested human blood specimens, indicating lack of PCR inhibition. When applied to 101 specimens negative for borreliae and 123 specimens found positive for *B. crocidurae* using combined 16S rRNA/*flaB*-gene PCR gold standard the observed Ct value for the clinical samples varied between 18 to 35. The multiplex real-time PCR yielded 100% specificity and 99% sensitivity (one positive specimen remained negative). No DNA remained from this false-negative specimen to enable targeted study of *glpQ* for mutations in the probe region. When applied to 101 specimens negative for borreliae and 30 specimens found positive for *B. recurrentis* and 18 specimens found positive for *B. duttonii* using gold standard, the multiplex real-time PCR yielded 100% specificity and sensitivity. As for *B. hispanica* DNAs samples, the two tick extracts were detected by *recC* probe.

## Discussion

In this study, all the negative controls remained negative in every real-time PCR experiment. Also, no evidence of PCR inhibition was detected using human blood as confirmed by amplification of the human albumin internal control in every PCR run. Specificity of primers and probes was confirmed by *in-silico* analyses and reinforced by experimental demonstrations that these assays failed to amplifyother microorganisms responsible for septicemia, including *R. felis* [21] and *T. whipplei* [22], all demonstrated to be emerging, highly prevalent pathogens in Africa and in Senegal in particular. Therefore, results reported herein were interpreted as authentic.

In this study, species-specific primers and probes based on the *glpQ, recC* and *recN* gene sequences were selected from the alignment of the *B. crocidurae, B. duttonii, B. recurrentis* and *B. burgdorferi* reference chromosome genomes [16], [17]. Plasmid sequences were avoided, because of their instability among different strains of the same species, and during replication of the same isolate, with a risk of resulting in false-negative results. This approach proved successful for the differentiation between *B. crocidurae, B. duttonii/B. recurrentis* and *B. hispanica*. Despite evident interest in distinguishing *B. duttonii* and *B. recurrentis* for accurate epidemiological purposes, discrimination between *B. duttonii* and *B. recurrentis* was not possible here in agreement with previously reported very close genetic and genomic proximity of both species [16], [17]. Indeed, genetic and genomic data suggested that *B. duttonii* and *B. recurrentis* could be regarded as a single *Borrelia* species [17]. This limitation may not be problematic as for the routine diagnosis since these two species are respectively transmitted by tick and lice in very different epidemiological contexts [23]. Also, the multiplex real-time PCR proved highly sensitive, detecting 100 copies, that is more sensitive than the 10^3^–10^5^ borreliae/µL reported for microscopy [14], [24]. Previously, borreliae not detectable by microscopy, were detected by using real-time PCR targeting the *fla* and the *glpQ* genes [3], [15]. These assays however could not identify borreliae at the species level [3], [15]. Another real-time PCR assay was devoted to the specific detection of *B. recurrentis* and *Rickettsia prowazekii*, as these two pathogens are both transmitted by body lice. This targeted the flagellin gene of *B. recurrentis* with a sensitivity of 10^1^ borreliae [25].

The developed real-time PCR was validated against large number of samples from areas endemic for diverse borreliae causing human infection in Africa, showing 100% sensitivity and specificity except for *glpQ* gene which had 99% sensitivity due to failure to identify *B. crocidurae* in one blood specimen. Unfortunately, we could not further analyze this specimen to assess whether this false negative result arose from *glpQ* mutations or was indicative of a different species or subspecies related to *B. crocidurae*. Indeed, several recent reports indicate that new relapsing fever *Borrelia* species are present in Tanzania and South Africa [26], [27]. Therefore careful optimization is required to ensure that the multiplex real-time PCR technique employed will not miss these new species or other *Borrelia* species. For this, we incorporated the 16S rRNA gene probe in the system to serve as an indicator of the detection of any relapsing fever *Borrelia* in the specimen. A specimen detected positive by the 16S rRNA gene probe and negative by the species-specific probes would indicate a new *Borrelia* species. Such samples could be further subjected to *in situ* typing such as the recently described multiple spacer sequence typing [16].

In conclusion, as detection and identification of these genetically closely related relapsing fever borreliae in Africa remains challenging, the multiplex real-time PCR assay reported herein offers significant improvement over existing procedures for the diagnosis of relapsing fevers in Africa. Importantly, it permits rapid differentiation of relapsing fevers from the clinically similar malaria, that requires drastically different therapeutic management. It is a sensitive and specific technique capable of detection of major *Borrelia* pathogens in humans, yet will not overlook detection of potentially new species. This multiplex real-time assay being amenable to point-of-care laboratories in Africa [28], it provides an effective solution for enhanced characterization of relapsing fever borreliae in Africa, improving medical management of patients and facilitating epidemiological studies.

## Supporting Information

File S1 **STARD checklist.**
(DOC)

File S2 **STARD flowchart.**
(PDF)
