# Supplementary figures and images for: Multiplex Real-Time PCR Diagnostic of Relapsing Fevers in Africa
Source: PLoS Negl Trop Dis. 2013 Jan 31;7(1):e2042. doi: 10.1371/journal.pntd.0002042 (PMC3561136; doi:10.1371/journal.pntd.0002042)

General example

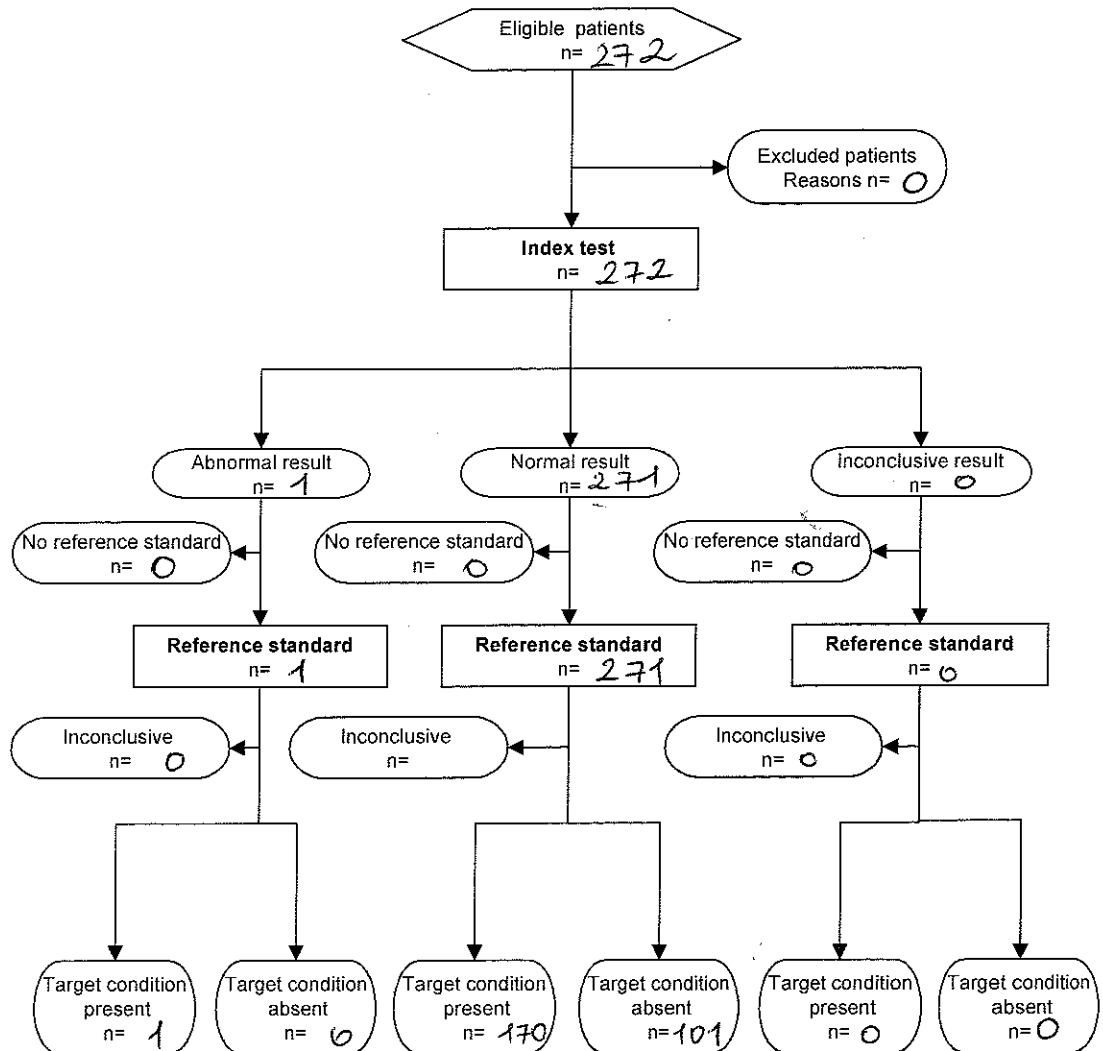

Supplement: File S2 — STARD flowchart. (PDF) [file pntd.0002042.s002.pdf]
